# Morbidity patterns among the welders of eastern Nepal: a cross-sectional study

**DOI:** 10.1186/s40557-016-0151-y

**Published:** 2016-11-15

**Authors:** Shyam Sundar Budhathoki, Suman Bahadur Singh, Surya Raj Niraula, Paras K. Pokharel

**Affiliations:** School of Public Health & Community Medicine, B P Koirala Institute of Health Sciences, Dharan, Nepal

**Keywords:** Welders, Morbidity among welders, Occupational health and safety in Nepal

## Abstract

**Background:**

Welding process has many hazards that the welders are exposed to resulting in numbers of health effects and diseases. Safety measures and practices among welders are important ways of preventing or reducing the health hazards associated with this occupation. We conducted this study to find out the morbidity patterns among the welders working in eastern Nepal.

**Methods:**

A cross sectional study was conducted among 300 welders using semi structured questionnaire. Morbidity categories were classified based on symptoms experienced in past 6 months.

**Results:**

All the welders learned welding by apprenticeship, without any formal health and safety training. Injury was the most common problem at work followed by skin problems and eye symptoms. Age of the welders, duration of employment & welding hours per day were associated with the morbidities among the welders.

**Conclusions:**

There is a need for occupational health services for welders in Nepal. While further research may be required to make policy recommendations, the current study provides a baseline morbidity burden among these welders to look for interventions to promote health and safety at work for this neglected group of workers in Nepal.

## Background

Welding is regarded as a hazardous profession where the welders are exposed to heat, burns, radiation, noise, fumes, gases, electrocution, and even the uncomfortable postures involved in the work; the high variability in chemical composition of welding fumes, which differs according to the work place, method employed, and surrounding environment; and the routes of entry through which these harmful agents enter the body [1]. Common health effects in the eyes, lungs, skin and fertility are reported among the welders [1, 2]. The employment of safety measures to minimize the hazards and the use of personal protective equipment has been seen to decrease morbidity and mortality among the welders. There is low awareness of hazards and use of protective equipment among welders around the world [3, 4] and Nepal [5].

In Nepal, the Department of Labor and Employment Promotion established under the Ministry of Labor and Transport Management, in 1971, is responsible for occupational safety, health and working conditions. There is no separate section or branch for safety and health in the ministry of health. The safety and health provisions under the Labor Act, 2048 (1992), are enforced by the Factory Inspectors of Labor Office [6].

Limited studies can be found in occupational health [7–12] and among welders [5] in Nepal. The concept of occupational health and safety is relatively new and very few industries maintain optimum occupational standards even to the oldest industry of Nepal with only a few studies related to working conditions in industries, have been conducted so far [11]. While welders are exposed to hazardous working environment and the employment of safety measures along with the use of PPE is low, it is imperative to find out the existing health problems among these welders [5].

In Eastern region of Nepal, workplaces of welders are usually located around mechanic workshops, motor spare-parts markets and along major highways of cities, where they establish privately owned small-scale workshops. While health effects and conditions are reported from welders around the world, little is known about the health of the welders in Nepal. Thus we conducted this study to find out the morbidity patterns among the welders in eastern Nepal in order to generate evidence for further studies and policy advocacy.

## Methods

### Study setting and design

We conducted a community-based cross-sectional study in Sunsari, Morang and Jhapa Districts of Eastern Nepal. Welders are found in the metal workshops, which are usually located in the cities (the cities being an industrial area), around main road centers and along the sides of highway. The metal workshops have welding spaces that are out doors with a shed to protect from sunlight and rain.

### Sample size & sampling technique

This research is a part of the health status of welders in Eastern Terai of Nepal study conducted from April to July 2011. Sample size was estimated using formula (Z_1-α_)^2^pq/L^2 ^where prevalence of injuries (p) was taken as 37.7 %, from the study in Nigeria [13]. A total of 300 welders working in eastern Nepal were taken into the study. The study employed a cluster sampling method taking each metal workshops as a cluster. The detail calculation of the sample size and the sampling technique has been described in the study published in 2014 [5]. Welders with work experience more than 1 year were included in the study and if they did not give consent or were not able to meet after two visits, then were considered as non-respondents.

### Data collection

Data was collected by interview technique using pre-tested questionnaires prepared by expert consultation and literature review. The questionnaire included questions pertaining to age, gender, marital status, education, income, duration of employment in years, exposure and morbid conditions of the study participants. Exposure was calculated in hours per day. Self-reported symptoms experienced in past 6 months were recorded and classified according to the categories as defined in the operational definitions below.

### Operational definitions

#### Arc eye (Photokeratatitis) related symptoms

The symptoms are foreign body sensation, eye irritation, eye ache, photophobia, blurred vision, watery eyes occur within few hours of welding. The welder positive for all the above symptoms was classified in this category [14].

#### Metal Fume Fever (MFF) related symptoms

This category comprised of at least one symptoms of fever, feelings of flu, general malaise, chills, dry cough, metallic taste, and shortness of breath, at least 3–10 h after exposure to welding fumes and the symptoms resolving in 24 to 48 h. Dry cough was a throat symptom that distinguishes MFF from Asthma symptoms [15].

#### Asthma related symptoms

Presence of cough, wheezing or chest tightness, experienced by a welder during or within few hours of welding are classified in this category [15].

#### Hearing problem related symptom

The reported symptoms of decreased hearing was categorised in this category.

#### Skin problems related symptom

The reported symptom of erythema and skin irritation of the skin was categorised in this category.

#### Musculoskeletal problems related symptoms

The reported symptoms of low back pain, muscle ache was categorised in this category.

#### Injury

Any cuts, abrasion, burns, and blunt trauma occurring at work among the welders was reported in the injury category in this study.

#### Poverty line

As defined by World bank as income of 1.25 USD per day and used for developing countries [16].

### Statistical analysis

Data was entered in MS-Excel 2007 software and the Statistical Package for Social Sciences (SPSS, version 17) was used for data analysis. Frequencies and other descriptive statistics were summarized using frequency distribution tables and a two way table showing duration of employment and working hours with different morbid conditions. *T*-test and Mann Whitney-*U* test was used to compare two means for age of the welders, duration of employment status and working hours of the participants with morbid conditions depending upon nature of data. Multiple logistic regression was performed for the variables that were significant in the bivariate analysis. The probability of significance was set at 5 % level of significance and 95 % Confidence limit.

### Ethics, consent and permissions

The approval for this study (Ref: Acd-628/069/070) was taken from the Institutional Ethical Review Board (IERB) of the B P Koirala Institute of Health Sciences, Dharan, Nepal. Informed consent was taken from the participants before the study.

## Result

Total of 300 welders from Jhapa, Morang and Sunsari districts participated in this study. The study showed that 100 % of the welder started the job as an apprentice to an experienced welder. They learned welding skills through hands-on apprenticeship. All welders (300) had not been oriented on basic first aid at work through orientations or trainings. The socio-demographic characteristics of the welders can be found in Table 1.

**Table 1:** Distribution of the welders according to the socio-demographic characteristics (*n* = 300)

Socio-demographic characteristics		Number of welders	Percent
Age (years)	<20	14	4.7
	20–29	113	37.7
	30–39	144	48
	40–49	29	9.7
Marital status	Unmarried	40	13.3
	Married	256	85.3
	Divorced/widowed	4	1.3
Educational status	Illiterate	21	7
	Primary school	111	37
	Secondary school	145	48.3
	Higher secondary school	23	7.7
Income per capita per day	Below poverty line	243	81
	Above poverty line	57	19
Duration of employment (years)	1–5	166	55.3
	6–10	85	28.3
	≥11	49	16.3

### Socio-demographic characteristics of the welders

More than 85 % of the welders are between the ages of 20 to 39 years with almost half of the welders in the category of 30–39 years. The education level attainment of the welders show almost half (48.3 %) of the respondents have completed up to the Secondary school, followed by 37 % up to the primary level education and showing that 93 % of the welders in this study were literate. Most of the respondents (81 %) were in the category below the poverty line.

It was seen that majority of welders (55.3 %) were employed for a duration of 1–5 years. The mean duration in years of employment of the welders in the study population was 6.94 years (Mean ± SD: 6.94 ± 5.30) ranging from a minimum of 1 year to a maximum of 25 years. The mean duration in hours per day spent in welding was 4.18 h (Mean ± SD:4.18 ± 0.96 h) with a variation of a maximum of 6 h to a minimum of up to 2 h of welding per day. The median hour of welding however was 4 h.

It was seen that about 90.7 % of morbid conditions was related to injuries during working hours followed by skin problems (74.3 %) as shown in Table 2.

**Table 2:** Distribution of morbidity conditions among welders (*n* = 300)

Morbidity conditions	Numbers^a^	Percentage^a^
Hearing problems	107	35.7
Metal Fume Fever	130	43.3
Musculoskeletal problems	140	46.7
Asthma Related Problems	140	46.7
Arc Eye related problems	184	61.3
Skin Problems	223	74.3
Work related Injury	272	90.7

^a^multiple responses

Table 3.shows that age of the welders, mean duration of employment in years and daily welding hours was found to be statistically significant for arc eye related symptoms, metal fume fever related symptoms, asthma related symptoms, skin problems related symptoms and hearing problems related symptoms. However, musculoskeletal problems related symptoms and injuries were found to be insignificant.

**Table 3:** Comparison of Morbidity categories with age of the welders, duration of employment and daily welding hours (*n* = 300)

Morbidity categories		Number (%)	Age of the welders (years)		Duration of employment (years)		Welding Hours daily (Hours)	
			Mean ± SD	*p*-value*	Mean ± SD	*p*-value**	Mean ± SD	*p*-value*
Arc eye related symptoms	Yes	184 (61.3 %)	33.77 ± 5.93	**<0.001**	8.84 ± 5.59	**<0.001**	4.45 ± 0.91	**<0.001**
	No	116 (38.7 %)	27.35 ± 5.55		3.92 ± 2.89		3.76 ± 0.87	
Metal fume fever related symptoms	Yes	130 (43.3 %)	32.59 ± 6.41	**0.003**	8.21 ± 5.80	**<0.001**	4.42 ± 1.01	**<0.05**
	No	170 (56.7 %)	30.29 ± 6.53		5.96 ± 4.67		4.01 ± 0.88	
Asthma related symptoms	Yes	140 (46.7 %)	32.66 ± 6.85	**0.001**	8.11 ± 5.82	**<0.001**	4.38 ± 0.96	**<0.05**
	No	160 (53.3 %)	30.09 ± 6.09		5.91 ± 4.56		4.01 ± 0.92	
Skin problems related symptoms	Yes	223 (74.3 %)	32.28 ± 6.63	**0.000**	7.88 ± 5.58	**<0.001**	4.31 ± 0.94	**<0.001**
	No	77 (25.7 %)	28.43 ± 5.53		4.19 ± 3.03		3.81 ± 0.88	
Hearing problems related symptoms	Yes	107 (35.7 %)	36.55 ± 5.70	**0.000**	10.62 ± 6.28	**<0.001**	4.73 ± 0.85	**<0.001**
	No	193 (74.3 %)	28.37 ± 5.03		4.90 ± 3.20		3.88 ± 0.87	
Musculoskeletal problems related symptoms	Yes	140 (46.7 %)	30.96 ± 5.18	0.412	6.24 ± 3.45	0.882	4.21 ± 0.95	0.688
	No	160 (53.3 %)	31.58 ± 7.59		7.55 ± 6.44		4.16 ± 0.96	
Injuries	Yes	272 (90.7 %)	31.30 ± 6.60	0.949	7.04 ± 5.42	0.619	4.18 ± 0.96	0.847
	No	28 (9.3 %)	31.21 ± 6.37		5.42 ± 3.89		4.21 ± 0.87	

* = *t* test, ** = Mann Whitney *U* test, Bold indicates *p*-value < 0.05, taken as significant

In multiple logistics regression, age of the welders, duration of employment and welding hours per day were all associated with arc eye related symptoms; welding hours per day was associated with metal fume fever related symptoms; duration of employment was associated with skin problems related symptoms; and age of the welders and welding hours per day was also associated with hearing problems related symptoms (Table 4).

**Table 4:** Association of the selected variables with the morbidity categories among the welders

Variables	Age (years)			Duration of Employment (years)			Welding Hours per day (hours)		
	AOR	CI	*p*-value	AOR	CI	*p*-value	AOR	CI	*p*-value
Arc eye related symptoms	1.086	1.005–1.174	**0.037**	1.220	1.085–1.371	**0.001**	1.544	1.097–2.173	**0.013**
Metal fume fever related symptoms	0.982	0.920–1.048	0.587	1.078	0.995–1.168	0.068	1.408	1.060–1.871	**0.018**
Asthma related symptoms	1.009	0.946–1.077	0.785	1.055	0.974–1.143	0.187	1.292	0.975–1.712	0.075
Skin problems related symptoms	0.964	0.892–1.042	0.362	1.259	1.109–1.431	**0.000**	1.404	0.987–1.999	0.059
Hearing problems related symptoms	1.326	1.196–1.471	**0.000**	1.024	0.917–1.143	0.675	1.919	1.315–2.802	**0.001**

Bold indicates *p*-value < 0.05, taken as significant

## Discussion

All the welders were male. Males tend to engage in hazardous work, which is seen in this study. This finding has also been reflected by a study in Nigeria [13] and India [17] where welders are predominantly males. The younger age group involvement suggests the early age apprenticeship through hands on training from experienced welders at the start of the job. It was found that about 85.3 % respondents were married, a finding similar in India [18].

This study showed 93 % of welders’ population in this area have had education, similar to a study conducted in Northern Ethiopia [19, 20]. The Census 2001 [21] shows the literacy rate of 54.1 % with Literacy rate among men being 65.5 %. The Nepal Demographic Health Survey 2011 [22] shows 89.2 % of the men in the eastern terai have had some levels of education. This could be a part of the reason for 93 % of the welders having education in the same region. The level of education among the welders showed the 48.3 % of them have completed Secondary school. The reason for not continuing the education over primary education by the 37.7 % could be related to need for financial support of own self and family and a very few who did not find it important to continue further.

Eighty one percent of the welders from this study were living below the poverty line. The national data [23] shows 22 % of the population living below the national poverty line. This gap may need to be explored, despite being employed the welders found below the poverty line.

Less than a fifth of the welders were working for more than 10 years. The mean duration of experience of welders in this study is 6.94 years. The working population in this profession has high turnover in this area with very little people with experience still working here. The welders may have learnt welding and, moved to bigger cities for better wages. However studies in Nigeria [13], shows 74.8 % welders with experience of more than 10 years including 24.7 % of welders with experience of more than 21 years and mean experience of 15.6 years in Nigeria and 81.8 % welders working for 10 years and more including 25, 9 % in 20–29 years and 22.8 % in 30 and above category with mean experience of 20.33 years in Canada [24].

The mean welding hours per day in this study was 4.18 (±0.95) hours with a minimum duration of 2 h per day and a maximum of 6 h per day. In studies from Nigeria [3] and India, [17], the welders had working hours up to 12 h a day with more than 90 % of welders having working hours of more than 6 h up to 12 h. One reason for less duration of hours of welding in our study may be the long hours of power outage in our country [25]. Welders sometimes also performed tasks like cutting, hammering but not a regular basis.

In this study, all welders had one or more complaints on health, which was similar to findings from Nigeria [13], where 96.4 % welders had one or more health complaints. The most common complaints were injuries (90.7 %) due to physical hazards including cuts by sharp metals, blunt force injury and fall injury. Complaints of injuries among Nigerian welders [13] was 37.7 %. This difference could be due difference in the use of PPE and the duration of employment among the welders in the two settings [13].

After injuries, other common self-reported symptoms were skin problems (74.3 %), and arc eye related problems (61.3 %), asthma problems (46.7 %) while the least common problem was hearing problems (35.7 %). Skin, eye, and respiratory related morbidity are some of the common health hazard related in several studies [13, 17, 26, 27]. Arc eye related symptoms in this study were found among 61.3 % of welders. Arc eye related problems was seen in 75.7 % among the welders of Benin [13]. In a study done among Canadian welders, ocular symptoms were reported by 53.3 % of welders by El-Zein [24].

Metal fume fever related symptoms in this study (43.3 %) is similar with Isah [13] finding of 43.8 %. Likewise, the prevalence of arc eye symptoms was reported to be 36 % in another study in Ethiopia [4]. While our study showed that asthma related symptoms were seen among 46.7 %, a finding that mirrors that of a study by Jani [28] where respiratory morbidity was found to be 44.4 % among welders in India and El-Zein [24] reported 40.5 % among welders in Canada.

Hearing problems (12.4 %) in our study was similar a study conducted by Tadesse [4]. The morbidities among welders in this study seems high, which may not be surprising, considering that only 47.7 % of these welders in the study used at least one type of PPE. The awareness of hazards and use of PPE among these welders has been published in 2014 [5].

Hearing complaints have been reported least in our study population. There could be several reasons for this. Welders with severe hearing complaints may have left this job. The other reason could be the fact that these workers were usually working in outdoor open space resulting in mixture of noise from vehicles. Also, we had not used any objective criteria for auditory assessment such as audiometry, which would have enabled us to diagnose at least mild to moderate hearing loss.

These differences in the prevalence rate of self -rated occupational morbidities could presumably be due to the differences in socio-economic levels, lack of safety training and awareness; and limited occupational safety and health services and practices [5].

Arc eye (*P* < 0.001), Metal Fume Fever (*P* < 0.001), Asthma (*P* < 0.001), Skin problems (*P* < 0.001), Hearing problems (*P* < 0.001) and Musculoskeletal problems (*P* < 0.01) were all seen significantly associated with age of the welders, duration of employment and welding hours per day. This showed that symptoms of the welding related problems are seen more in the more aged welders, welders working for more number of years and welders working for more number of hours per day. These findings coincide with the findings by Isah [13]. Studies done by Sharifian [29], Stoleski [30], Jani [28] and by Erhabor [31] also showed that with increase duration of exposure (years), symptoms were likely to increase showing an association. In another study in India both morbidities were found to be significantly associated with years of employment among welders than non -welders [32].

Injuries were not found to be associated with age of the welders, duration of employment and welding hours per day. Although studies have been conducted with regard to morbid conditions among welders, limited literatures were found to assess association of welding hours with morbid conditions. A study in South India [33] also found no association between injuries with duration of employment and welding hours per day. The reason for no association with age of the welders, employment duration and working hour per day, could not be explained in this study. However a possible reason could be under reporting of the injuries especially of the minor cuts and abrasions which may be ignored. All welders are daily wage workers and any absence from work may lead to no income for that day. So welders may tend to not report minor symptoms. With majority (81 %) of respondents living below poverty line, affordability to healthcare centre for injuries of any kind seems expensive. As for musculoskeletal problems, most of the participants were observed to be spending their working time sitting. This finding has an important implication because welders are involved in sedentary occupation for years and prolonged sitting (usually in squatting position) or standing have been found to be related to high incidence of low back pain [34].

### Limitations of the study

This study did not use any objective instruments (audiometry, spirometry etc.) to measure the welding related health problems. The use of self-reported symptoms may have under/overestimated the welding related health problems in this study. We attempted to address this issue by using operational definitions. The welding hours are short in this study, the electricity supply in the in the study area was 4–6 h/day only. In Nepal we have country frequent power cuts for up to 16 h in a day [25]. We could not quantify the time the welders may have contributed to other physical works of cutting and hammering which could also contribute to symptoms. However, it was noted that welders are not involved in other physical tasks regularly.

## Conclusion

Welders in this study have symptoms of multiple morbidities in huge proportion that could be related to welding. Evidence from around the world have already identified welding as a hazardous profession and the welders have suffered serious conditions due to welding. This is probably among the first evidence on morbidities associated with welding in Nepal. Based on the findings from this studies further researches will be needed to identify ways to decrease the morbidities by reducing hazards at work and increasing PPE use at work by welders in Nepal.

## Abbreviations

MFF: Metal fume fever
PPE: Personal protective equipment
